# Integrating sex and gender into biomedical research requires policy and culture change

**DOI:** 10.1038/s44294-024-00027-x

**Published:** 2024-07-02

**Authors:** Alice Witt, Marina Politis, Robyn Norton, Kate Womersley

**Affiliations:** 1 The George Institute for Global Health, London, UK; 2 Imperial College London, London, UK; 3 UNSW Sydney, Sydney, NSW, Australia

**Keywords:** Physiology, Diseases, Health care

## Abstract

Most biomedical, health and care research does not adequately account for sex and gender dimensions of health and illness. Overlooking and disregarding the influence of sex and gender in research reduces scientific rigour and reproducibility, which leads to less effective treatments and worse health outcomes for all, particularly women and sex and gender diverse people. Historically, there has been minimal sex and gender policy innovation in UK medical research. To address this, stakeholders from across the UK research sector have been collaborating since spring 2023 to co-design a sex and gender policy framework to be implemented by research funders, as part of the MESSAGE (Medical Science Sex and Gender Equity) project. In the first Policy Lab, held in London in May 2023, 50 participants, including representatives from funding organisations, medical journals, regulators, clinicians, academics and people with lived experience, identified two key priorities for future action: 1) A whole system approach to policy change, and 2) Technical capacity-building and wider culture change efforts. In pursuing these priorities and collaborating cross-sectorally, UK stakeholders are engaged in an internationally innovative approach aimed at realising sustainable and impactful sex and gender policy change. Drawing on MESSAGE Policy Lab discussions, we set out key actions needed for the UK research sector to embed meaningful accounting for sex and gender as a new norm for research practice.

## Integrating sex and gender into biomedical research requires policy and culture change

Historically and to this day, sex and gender considerations have not been adequately accounted for in biomedical, health and care research. This persists across data collection, analysis and reporting of findings in both bench and clinical research. A 2019 study found that just 49% of pre-clinical research studies reported using both male and female research subjects, and only 42% analysed their data by sex^1^. In clinical medicine, just 22% of phase I trial participants are female^2^, with 67% of publications reporting data by sex^3^. Sex and gender data gaps in the evidence base reduce scientific accuracy and generalisability, leading to less effective treatments, increased risks of patient harm and worse outcomes. For example, of the ten drugs withdrawn from the US market between 1997 and 2000, eight posed a greater health risk to women than men due to trials having poorly accounted for sex and gender dimensions^4,5^. Though these gaps predominantly impact women, girls and sex and gender diverse people, addressing sex and gender gaps in the evidence base and research practice will improve health outcomes for all people.

### Research funder policies are an important lever for initiating change

Medical research funders around the world, most notably the Canadian Institutes of Health Research (CIHR), the National Institutes of Health in the US and Horizon Europe, have adopted policies to address sex and gender data gaps^6^. An evaluation of the CIHR policy (2010) showed that such interventions can have a considerable impact: in the first decade of the policy’s implementation, the proportion of funding applications that considered sex rose from 22% to 83%, and of those considering gender increased from 12% to 33%^7^. Since 2015, the number of Canadian research publications that account for sex and/or gender rose by 64%^8^. By contrast, in the UK there is no unified sex and gender guidance for researchers, and only one funder has a sex and gender policy – the Medical Research Council’s *Embedding diversity in research design*, which was launched in 2023^9^. UK research funders, however, have now shown an appetite for change. Over 30 stakeholders have publicly stated their support for sex and gender policy action since December 2023, including major government and charitable funders^10,11^. Over the past 12 months, organisations across the UK biomedical, health and care research sector have engaged in co-designing a policy framework for research funders to improve how research accounts for sex and gender dimensions. Led by The George Institute for Global Health’s MESSAGE (Medical Science Sex and Gender Equity) project, the co-design process took place over three Policy Labs in 2023–2024 (www.messageproject.co.uk).

At the first Lab, held in May 2023, 50 representatives from the UK biomedical, health and care research sector came together to set out a vision for policy efforts to improve how researchers account for sex and gender dimensions^12^. The group comprised representatives from research funding organisations, regulators and academic publishers, people with lived experience, researchers, clinicians and government officials. The Lab identified two key priorities for enhancing the impact of policy action:

1. Sex and gender policies should be designed and delivered through a whole system approach.
2. Technical capacity-building and culture change across the research sector is needed to support policy implementation.

### A new norm for research practice to improve scientific rigour and reproducibility

Sex and gender are relevant to the vast majority of biomedical, health and care research questions, and accounting for these dimensions is an essential component of conducting robust, rigorous and reproducible science. Despite this, identifying potential sex and gender differences and similarities – rather than merely controlling for sex or gender – is currently considered a niche area of expertise, rather than a standard that ought to be widely practised. Policy interventions are vital for bringing about a paradigm shift in how the research community thinks about, conducts and values research, to centre explicit accounting for sex and gender dimensions as a core part of high-quality research practice.

To enhance scientific rigour and reproducibility, researchers must embed sex and gender considerations in their thinking at every stage of the research cycle, from study design and data collection to analysis and reporting of findings^13,14^. Improved representation of all sexes and genders in research is a critical first step to address historic biases in research inclusion. Representation must be paired with a commitment to disaggregate data by sex and/or gender when analysing and reporting findings, which is rare in current practice^15,16^. Accounting for sex and gender does not mean powering every study to identify statistically significant sex and/or gender differences, but rather designing studies in a way that allows possible sex and/or gender dimensions of health and disease to emerge in study data and inform future research. Simply reporting sex- and gender-disaggregated data will widen the collective knowledge of the research community, enabling future research, including meta-analyses, to study important sex and gender differences in more depth.

Most importantly, greater transparency is needed in published research about the sex and/or gender characteristics of the study sample, whether data analysis has accounted for these variables, and whether the way sex and/or gender has been accounted for is a strength or limitation of the study. This change in convention will have considerable impact by bringing clarity to the existing evidence base and highlighting data gaps for further research.

### Sex and gender policies should be designed and delivered through a whole system approach

MESSAGE Policy Lab participants identified that a whole system approach is needed to change expectations for what high-quality research includes and how it should be evaluated. A coordinated and consistent approach between the many organisations that make up the medical research sector will ensure that new requirements are clear to researchers and are as easy as possible to adopt.

The group identified that funder policies should be the first step in this process because they shape researchers’ thinking at the first stage of study design. Once funder requirements are in place, regulators and publishers will be able to reflect those requirements in their guidance over time, as researchers become more familiar with and skilled at accounting for and reporting sex and gender considerations. Policy Lab participants stated that a joint roadmap and timeframe for sector-wide policy roll-out would support cohesion between the different organisations.

Changes in how sex and gender are considered in research requires investment of resources, particularly by funders, to upskill researchers and to address gaps in the existing knowledge base. Internally, organisations must dedicate time to considering how sex and gender will be integrated in existing funding application review systems and must appoint dedicated members of staff to be responsible for policy implementation and evaluation. Lab participants emphasised that a whole system approach would help to reduce the burden of additional work by enabling actors to share and collaborate on strategies and resources.

### Technical capacity-building and culture change across the research sector is needed to support policy implementation

The second priority outlined by Policy Lab participants was the need to provide clear guidance from the outset to support researchers to meet new policy expectations. Guidance should raise awareness about the relevance of sex and gender considerations to biomedical, health and care research, and provide researchers with the skills to implement these changes in their work. The fact that many published papers use the terms sex and gender interchangeably underscores the need to build researchers’ knowledge about the differences between usage of the two terms and their distinct relevance in the context of biomedical, health and care research^17,18^. Supporting researchers to reflect on which term is most relevant to their research question will enable them to identify which research participants or subjects they need to include to develop an appropriately representative sample. Guidance on statistical considerations and best practice for procurement, recruitment and retention of a more diverse sample will also be important.

Wider advocacy efforts will be needed to bring sex and gender considerations to the fore of research thinking and to establish a new norm of transparency around reporting of sex and gender dimensions. A crucial component of this will be addressing misconceptions which have historically been used to justify the exclusion of female subjects and participants, such as the (disproven) contention that female hormonal variability throughout the oestrus cycle might obscure results^19,20^. Likewise, women’s reproductive capacity has historically been used to justify their exclusion from drug trials, in large part due to the thalidomide scandal of the 1960s, which also damaged trust with the public^21^. Awareness-raising efforts must highlight that excluding women from clinical trials only to expose them to those very medicines and treatments outside of the comparatively safe laboratory environment increases the risk of patient harm. Moreover, concerns about reproductive capacity can now be addressed by modern contraception methods and do not apply to women who are post-menopausal.

Showcasing and rewarding examples of best practice in considering sex and gender will be important to establish role models, incentivise new ways of working, and attract further investment to fill knowledge gaps. Increasing the sex and gender diversity of the research workforce, and particularly of those in senior positions, will support this culture change^7^.

### Accounting for sex and gender will generate new opportunities for research impact

Improved accounting for sex and gender in biomedical, health and care research is essential for advancing the quality of scientific research. Funder policies will play an important role in initiating this process of change at the start of the research pipeline and, to embed this as a new norm for research practice, should be grounded in sector-wide collaboration and accompanied by capacity-building and culture change efforts. Enhanced integration of sex and gender considerations will trigger a virtuous cycle: as more researchers reflect on and describe the sex and gender characteristics of their study sample, increase the sex and gender equity of their research participants or subjects, and publish sex- and gender-disaggregated data, the evidence base will grow and strengthen. Reducing sex and gender data gaps in evidence will open new avenues for research, improve treatments and culminate in improved health outcomes for all.
